# Silencing erythropoietin receptor on glioma cells reinforces efficacy of temozolomide and X-rays through senescence and mitotic catastrophe

**DOI:** 10.18632/oncotarget.2937

**Published:** 2014-12-03

**Authors:** Elodie A. Pérès, Aurélie N. Gérault, Samuel Valable, Simon Roussel, Jérôme Toutain, Didier Divoux, Jean-Sébastien Guillamo, Marc Sanson, Myriam Bernaudin, Edwige Petit

**Affiliations:** ^1^ CNRS, UMR 6301-ISTCT, CERVOxy group. GIP CYCERON, Bd Henri Becquerel, BP5229, F-14074 CAEN, France; ^2^ CEA, DSV/I2BM, UMR 6301-ISTCT; ^3^ Université de Caen Basse-Normandie, UMR 6301-ISTCT; ^4^ Normandie University, France; ^5^ CHU de Caen, Service de Neurologie, Boulevard Côte de Nacre, F-14000 Caen, France; ^6^ Université Pierre et Marie Curie-Paris 6, Centre de Recherche de l'Institut du Cerveau et de la Moëlle épinière (CRICM), UMR-S975, F-75013 Paris, France; INSERM, U 975, F-75013 Paris, France; CNRS, UMR 7225, F-75013 Paris, France

**Keywords:** brain tumour, erythropoietin receptor, temozolomide, ionising radiation, senescence, mitotic death

## Abstract

Hypoxia-inducible genes may contribute to therapy resistance in glioblastoma (GBM), the most aggressive and hypoxic brain tumours. It has been recently reported that erythropoietin (EPO) and its receptor (EPOR) are involved in glioma growth. We now investigated whether EPOR signalling may modulate the efficacy of the GBM current treatment based on chemotherapy (temozolomide, TMZ) and radiotherapy (X-rays). Using RNA interference, we showed on glioma cell lines (U87 and U251) that EPOR silencing induces a G2/M cell cycle arrest, consistent with the slowdown of glioma growth induced by EPOR knock-down. *In vivo*, we also reported that EPOR silencing combined with TMZ treatment is more efficient to delay tumour recurrence and to prolong animal survival compared to TMZ alone. *In vitro*, we showed that EPOR silencing not only increases the sensitivity of glioma cells to TMZ as well as X-rays but also counteracts the hypoxia-induced chemo- and radioresistance. Silencing EPOR on glioma cells exposed to conventional treatments enhances senescence and induces a robust genomic instability that leads to caspase-dependent mitotic death by increasing the number of polyploid cells and cyclin B1 expression. Overall these data suggest that EPOR could be an attractive target to overcome therapeutic resistance toward ionising radiation or temozolomide.

## INTRODUCTION

Glioblastoma (GBM) are the most frequent primary malignant brain tumours in adults [1,2] but are still incurable despite current therapy including neurosurgery, alkylating agent based-chemotherapy with temozolomide (TMZ) and ionising radiation. Yet the survival median of GBM patients remains only around 15 months and the five-year survival is less than 10% [3]. Thus, both new therapeutic targets and improvement of treatments are needed. These tumours frequently exhibit hypoxia which promotes angiogenesis, invasion, glioma stem cell recruitment and tumour aggressiveness [4-6]. Hypoxia is likewise associated with poor response of GBM to radio- or chemotherapy [7-9]. The transcription factor Hypoxia-inducible factor-1 (HIF-1), one of the main regulators that orchestrates the cellular responses to hypoxia, is up-regulated in GBM and correlates with a poor patient prognosis [10-12]. It regulates the expression of a large number of hypoxia-responsive genes, including vascular endothelial growth factor (VEGF), the main regulator of angiogenesis that explains the intense vascular hyperplasia often seen in GBM. Thereby, therapies that target angiogenesis have generated substantial interest. In 2009, bevacizumab, a humanised monoclonal antibody against VEGF received accelerated approval from the US Food and Drug Administration for treatment of recurrent GBM but also as adjuvant treatments. However, the benefits of bevacizumab in combination or not with chemotherapy and radiotherapy are transient and tumours progress after only 3-5 months [13-18]. Thus, more efforts are yet required to develop effective therapeutics and overcome resistance to current treatments.

Recently, we and others have shown that erythropoietin (EPO), another major target gene of HIF activation, plays a role in tumour progression since GBM cells and glioma stem cells synthesise EPO and express its receptor [19-22]. We have shown, at the preclinical level, that EPOR silencing on glioma cells slows down the tumour growth through a proliferative arrest and we now speculate that EPOR signalling could modulate the sensitivity of glioma cells to chemo- or radiotherapy.

Because abrogation of the G2 checkpoint prevents cells from repairing DNA damage and forces apoptosis, it has emerged as an attractive therapeutic target to further improve the cytotoxic effects of anticancer therapies such as radiation or drugs [23-25]. More recently, G2 checkpoint has also been recognised to be crucial to impede mitotic death [26-28]. The importance of mitotic catastrophe as well as senescence is increasingly being recognised as tumour suppression mechanisms [29-31]. Permanently arresting tumour cells through the induction of senescence might improve anti-tumour agent efficacy. Indeed, cell senescence, originally defined as a proliferative arrest that occurs in normal cells is now considered as a general biological program of terminal growth arrest [32], which can be activated in tumour cells in response to DNA damage induced by chemotherapeutic drugs or radiation treatments [33-36]. The impact of accelerated senescence on the efficacy of chemo- and radiotherapy mainly depends on whether senescence arrest is followed by cell death including apoptosis, autophagy or mitotic catastrophe when the DNA damage response is inactivated or impaired. Mitotic catastrophe is a form of cell death that results from aberrant mitosis and leads to the formation of large nonviable cells with micronuclei. Rather than a true cell death mechanism, mitotic catastrophe might constitute an oncosuppressive pathway that can precede cell death [30,37-40]. Thus, mitotic catastrophe may end in apoptosis-like cell death when caspases are active or in a caspase-independent cell death leading to necrosis [41-45].

In this study, we show that EPOR down-regulation, by inducing G2/M cell cycle arrest and senescence of human glioma cells U87 or U251, improves the efficacy of radio- and chemotherapy, in both normoxic and hypoxic conditions. Silencing EPOR on glioma cells exposed to ionising radiation (X-rays) or chemotherapy (TMZ) enhances senescence and induces mitotic cell death, an effect that may be through an increase in cyclin B1 expression.

## RESULTS

### EPOR down-regulation induces G2/M cell cycle arrest in glioma cells

**Figure 1:**
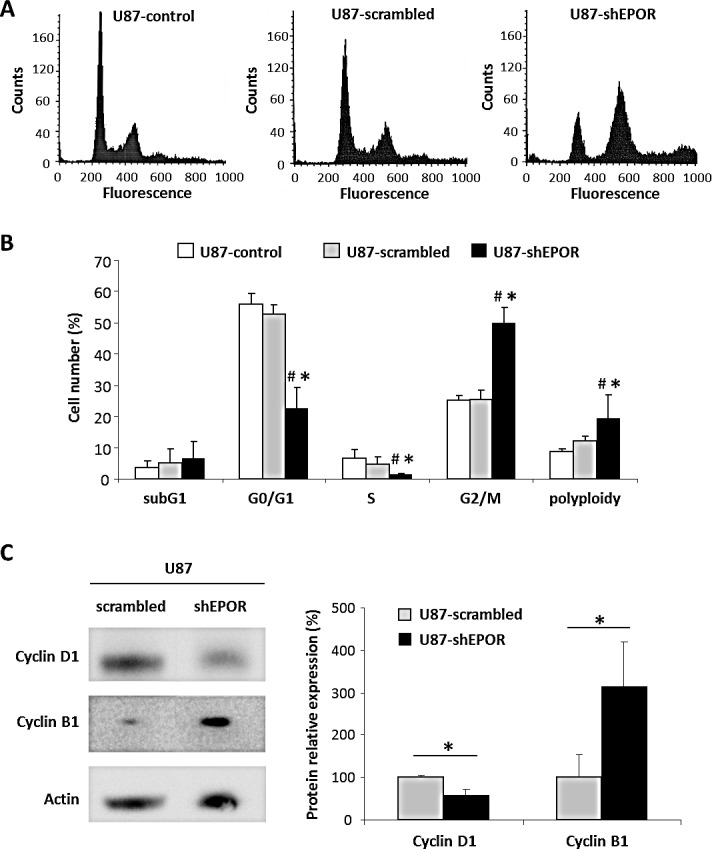
EPOR down regulation leads to a cell cycle arrest in G2/M phase and polyploidy At about 80% confluence, infected or not U87 cells were fixed and stained with propidium iodide to determine cell cycle status by flow cytometry or proteins of these cells were extracted to study by western blotting the expression of proteins involved in cell cycle progression. (A) Cell cycle profiles of U87-control, U87-scrambled and U87-shEPOR. (B) Quantification of the cell distribution in different phases of cell cycle. Mean ± SD, n=4 for each cell type; # p<0.05 control cells and * p<0.05 vs scrambled shRNA infected cells (Fisher's PLSD post-hoc test after a significant ANOVA). (C) Representative western blots on U87-scrambled and U87-shEPOR cells and quantitative analyses of cyclin D1, an important regulator of G1 to S phase progression, and cyclin B1 which is involved in G2/M cell cycle arrest. Mean ± SD, n=3 for each cell type; * p<0.05 vs scrambled shRNA infected cells (Student's *t*-test).

We previously showed that the inhibition of EPOR expression on U87 glioma cells leads to a decrease in their doubling time (Figure S1 supplementary data) and slows down the proliferation of U87 glioma cells *in vitro* as well as tumour growth *in vivo* [22]. We now show that EPOR silencing in U87 cells is associated with a cell cycle arrest in G2/M phase with a cell progression from a diploid to a polyploid state (Figure 1A) compared to U87-control and U87-scrambled cells. As presented on the Figure 1B, the proportion of U87-shEPOR cells arrested in G2/M phase (p<0.0001) as well as in polyploidy (p<0.05) is strongly increased (2-fold increase) whereas the cell number in G0/G1 (p<0.0001) and S (p<0.05) phases is significantly decreased relative to U87-scrambled or U87-control cells. We next checked whether the increase in the cell number in G2/M phase was linked to a G2 arrest and was not due to tetraploid cells in G1 phase. To this end, we verified that this increase persists independently of the cellular density (Figure S2 supplementary data) and we studied the level of cyclin B1 expression, used as a marker of G2 arrest, and cyclin D1 expression, as a specific protein of G1/S phase. Relative to U87-scrambled cells, we show that EPOR knock-down decreases the expression of cyclin D1 by 40% paralleled with a 210% increase in cyclin B1 (Figure 1C).

### EPOR down-regulation improves the efficacy of radiotherapy on glioma cells

**Figure 2:**
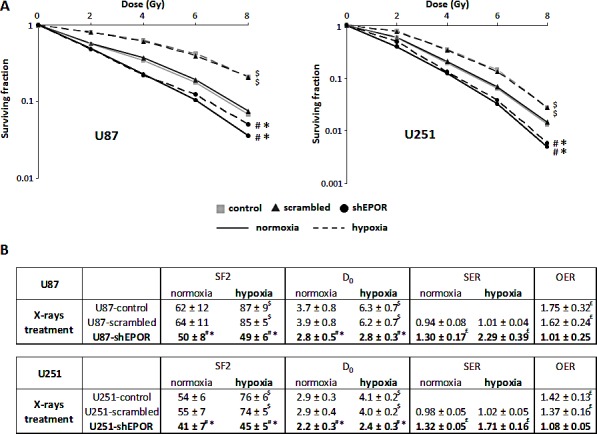
EPOR inhibition increases the sensitivity of glioma cells to ionising radiation and counteracts the hypoxia-dependant radioresistance The colony-formation assay was performed to determine the effects of various doses of X-rays (0, 2, 4, 6 and 8 Gy) on U87 and U251 cells in normoxic (21% O_2_) and hypoxic (1% O_2_) conditions. Colonies were counted 14 days and 21 days after radiation, for U251 and U87 cells respectively, to determine survival fraction (SF) and radiobiological parameters. (A) Effect of EPOR down-regulation on the response of U87 cells (left graph) or U251 cells (right graph) to radiation in normoxia and hypoxia. Mean ± SD, n=4 for each cell type; # p<0.0001 vs control cells in normoxic or hypoxic conditions, * p<0.0001 vs scrambled cells in normoxic or hypoxic conditions and $ p<0.0001 vs normoxia for each cell line (Fisher's PLSD post-hoc test after a significant two-way ANOVA (group and dose effects)). (B) Comparison of radiobiological parameters between control, scrambled and EPOR shRNA for the two glioma cell lines, U87 and U251, cultured in normoxic and hypoxic conditions. SF2=surviving fraction for 2 Gy; D_0_=mean lethal dose (dose for which SF is 37%); SER=sensitisation enhancement ratio (D_0_ control cells/D_0_ infected cells); OER=oxygen enhancement ratio (D_0_ hypoxia/D_0_ normoxia). Mean ± SD, n=4 for U87 and U251 cells. For SF2 and D_0_ parameters: # p<0.05 vs control cells in normoxic or hypoxic conditions, * p<0.05 vs scrambled cells in normoxic or hypoxic conditions and $ p<0.0001 vs normoxia for each cell line (Fisher's PLSD post-hoc test after a significant ANOVA). For SER and OER: £ p<0.05 vs theoretical value=1 (univariate t-test).

Since tumour cells in G2/M phase are known to be more radiosensitive [46,47], we next determined whether EPOR silencing affected the efficacy of radiotherapy on glioma cells. Hypoxia and p53 status of tumour cells are known to contribute to radioresistance [48-50]. Accordingly, we studied the radiosensitivity of glioma cells expressing or not EPOR in response to increasing dose of ionising radiation both in normoxic and hypoxic conditions. In addition to p53 wild-type U87 cells, we evaluated the response of p53 mutant type U251 cells to X-rays which express more strongly hypoxia-induced genes than U87 cells [51-53]. Radiation survival curves obtained from clonogenic assays (Figure 2A) show similar radiation sensitivity of U87-control and U87-scrambled cells in normoxia and, as expected, both cell lines display a radioresistance in hypoxia. Similar results are obtained with U251-control and U251-scrambled cells (Figure 2A). However, compared to the control cells, EPOR down-regulation on U87 or U251 glioma cells increases their sensitivity to radiation in normoxia (Figure 2A). Interestingly, in hypoxia, the increase in radiosensitivity is sustained and similar to that observed in normoxia for U87-shEPOR and U251-shEPOR cells. Radiobiological parameters estimated from the survival curves also reflect the greater harmful effect of X-rays on glioma cells in which EPOR expression was down-regulated (Figure 2B). For instance, for U251-scrambled cells, the surviving fraction at 2 Gy (SF2) is 55% in normoxia and rises to 74% in hypoxia, whereas the level of cell survival decreases to 41% and 45% for U251-shEPOR cells cultured in normoxia and hypoxia conditions, respectively. To assess and characterise the radiation-enhancing effects of EPOR inhibition on the glioma cells, the radiation dose required to reduce the survival fraction from 100% to 37%, namely D_0_ (mean lethal dose), was also calculated. D_0_ is considered as a measure of the intrinsic radiosensitivity of the cells. In normoxia, control, scrambled and shEPOR-infected U251 glioma cells exhibit D_0_ value of 2.9, 2.9 and 2.2 Gy respectively, and in hypoxia, D_0_ value of 4.1, 4.0 and 2.4 Gy (Figure 2B). Similar results are observed for the U87 cells (Figure 2B). This parameter also allows determining a sensitisation enhancement ratio (SER), calculated by determining the ratio of D_0_ of the control group versus infected glioma cells (SER>1 reflects a sensitisation to treatment). The increase in the SER for both U87-shEPOR and U251-shEPOR cells clearly indicates a radiosensitisation potential by inhibiting EPOR on glioma cells (Figure 2B). From D_0_ values, effects of oxygen on intrinsic radiation sensitivity can be also expressed quantitatively by the oxygen enhancement ratio (OER), which is defined as the ratio of D_0_ in hypoxia on D_0_ in normoxia. An OER value superior to 1 reflects a hypoxia-induced radioresistance, as it can be observed for U87-control cells (OER=1.75, p<0.05) and U87-scrambled cells (OER=1.62, p<0.05) as well as U251 cells (OER=1.42 and 1.37 for the U251-control and scrambled cells, respectively, p<0.05). However, the OER values of U87-shEPOR and U251-shEPOR indicate that EPOR knock-down counteracts the hypoxia-dependent radioresistance (Figure 2B). Collectively, these results provide evidence that EPOR is involved in the radiosensitivity of glioma cells not only in normoxia but also in hypoxia conditions.

### EPOR down-regulation increases the sensitivity of glioma cells to chemotherapy

**Figure 3:**
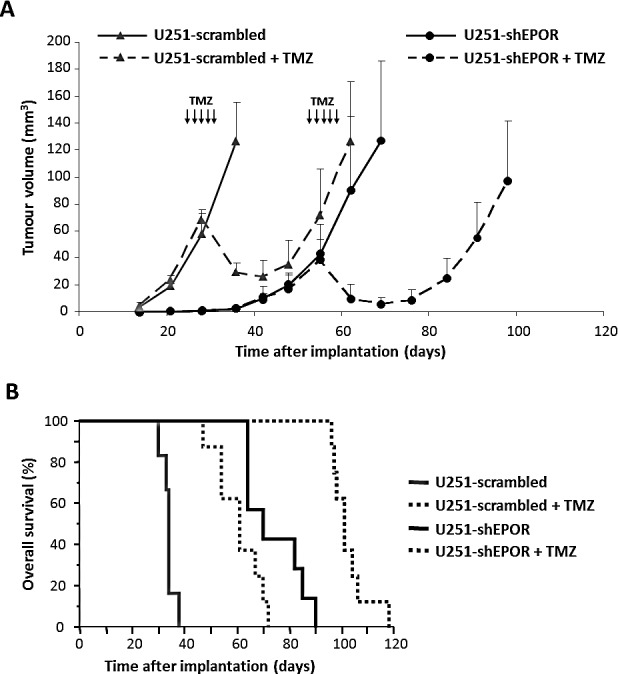
EPOR Inhibition expression on glioma cells potentiates the reduction of tumour volume and the increase of animal survival induced by temozolomide The orthotopic injection of U251-scrambled or U251-shEPOR cells was realised on *nude* mice and tumour progression was evaluated by MRI. At equivalent brain tumour volume (30-40 mm^3^), animals were treated *per os* by TMZ (10 mg/kg/day) during 5 consecutive days (D26-D30 for U251-scrambled and D54-D58 for U251-shEPOR). (A) Longitudinal MRI tumour volume follow-up of animals bearing U251-scrambled or U251-shEPOR tumours and treated or not with TMZ. MRI (T2w sequence) was done weekly to determine the tumour volume of each animal. The solid lines corresponds to untreated mice and the dotted lines shows mice treated with TMZ. Mean ± SD, n=6 mice for U251-scrambled untreated group, n=7 mice for U251-shEPOR untreated group and n=8 for U251-scrambled + TMZ and U251-shEPOR + TMZ groups. (B) Study of TMZ effect on animal survival by establishing the Kaplan-Meier curves for animals bearing U251-scrambled or U251-shEPOR tumours and treated or not with TMZ.

We then sought to investigate whether the EPOR inhibition on glioma cells could also modulate their chemosensitivity to TMZ. As presented on the Table 1, in normoxia, no significant difference in the TMZ toxicity is measured between control and scrambled-infected glioma cells. In contrast, EPOR silencing sensitises U87 or U251 cells to chemotherapy. The association of EPOR inhibition with TMZ treatment induces a greater decrease in glioma cell number as evidenced by the SER values of 1.78 and 2.44 for U87-shEPOR and U251-shEPOR cells respectively (Table 1). However, in hypoxia, the chemosensitivity is still observed for U87-shEPOR and U251-shEPOR cells, while hypoxia inhibits the toxicity of TMZ on the corresponding control cells. For each cell type, the chemoresistance induced by hypoxia might be illustrated by the OER (Table 1). The OER reaches a value superior to 1 for control and scrambled cells while it decreases to a value of respectively 0.99 and 0.73 for U87-shEPOR and U251-shEPOR cells, confirming the absence of TMZ drug resistance of glioma cells in hypoxic conditions when the EPOR expression is knock-down. We then sought to determine whether the TMZ chemosensitisation induced by the silencing of EPOR on glioma cells is retrieved *in vivo*. We used the U251 orthotopic model since in addition to hypoxia, this tumour model recapitulates most of the main pathological features described in human GBM [54-56]. As presented on Figure 3A, and according to our previous study [22], the extinction of EPOR on glioma cells slows down tumour growth by a factor two relative to control tumours. When the tumours reached an equivalent volume of 30-40 mm^3^ (i.e. U251-scrambled and U251-shEPOR groups were treated from 26 days and 54 days post-implantation, respectively), animals were treated or not with TMZ (10 mg/kg/day) for 5 consecutive days. A transient toxic effect of TMZ is observed for U251-scrambled group leading first to a significant decrease in tumour volume lasting 3 weeks post-treatment, but eventually followed by a tumour recurrence observed 55 days post-TMZ treatment. A similar kinetic profile of the tumour response to TMZ chemotherapy is observed for U251-shEPOR group (Figure 3A), but with a better efficacy. Indeed, the combination of EPOR inhibition with TMZ potentiates the cytotoxic effect of TMZ alone and this effect is maintained during 4 weeks post-treatment (p<0.0001) (Figure 3A). Therefore, 1 week after TMZ treatment, the tumour volume, calculated from MRI analyses, decreases by 57% for U251-scrambled tumours (p<0.0001) and by 77% for U251-shEPOR tumours (p<0.0001); with p=0.006 for U251-scrambled vs U251-shEPOR. In agreement with these results, the inhibition of EPOR on glioma cells greatly enhances the animal survival treated with TMZ. In response to TMZ, the median survival of U251-shEPOR animals is 101 days versus 61 days for animals bearing U251-scrambled tumours (Figure 3B). As depicted on Figure 3B, relative to untreated U251-scrambled group, the survivorship is increased by 79% for U251-scrambled TMZ group (p<0.0001), by 105% for U251-shEPOR untreated group (p<0.0001) and by 197% for U251-shEPOR TMZ group (p<0.0001), with p<0.0001 for U251-shEPOR TMZ group vs U251-scrambled TMZ group (Figure 3B).

**Table 1:** EPOR inhibition improves the response of glioma cells to temozolomide and reduces the hypoxiadependent chemoresistance TMZ treatment (25 μM) was performed in normoxic (21% O_2_) and hypoxic (1% O_2_) conditions on U87 and U251 cells and cell survival was determined at 72 hours post-treatment. The surviving fraction (SF= cell number after TMZ treatment/untreated cell number) was evaluated by counting the cell nuclei staining with Hoechst 33342. Mean ± SD, n=4; for SF: # p<0.05 vs control cells in normoxic or hypoxic conditions, * p<0.05 vs scrambled cells in normoxic or hypoxic conditions and $ p<0.0001 vs normoxia for each cell line (Fisher's PLSD post-hoc test after a significant ANOVA). For SER=sensitisation enhancement ratio (SF control cells/SF infected cells) and OER=oxygen enhancement ratio (SF hypoxia/SF normoxia): £ p<0.05 vs theoretical value=1 (univariate t-test).

U87		SF 25 μM (%)		SER		OER
		normoxia	hypoxia	normoxia	hypoxia	
TMZ treatment	control scrambled **shEPOR**	32 ± 5	78 ± 6 ^$^			2.52 ± 0.45 ^£^
		28 ± 8	76 ± 18 ^$^	1.17 ± 0.20	1.06 ± 0.21	2.84 ± 0.83 ^£^
		**22 ± 12**	**20 ± 10**^#^*	**1.78 ± 0.91**	**4.65 ± 2.07**^£^	**0.99 ± 0.39**

U251		SF 25 μM (%)		SER		OER
		normoxia	hypoxia	normoxia	hypoxia	
TMZ treatment	control scrambled **shEPOR**	51 ± 3	77 ± 6 ^$^			1.52 ± 0.07 ^£^
		54 ± 13	75 ± 17 ^$^	0.98 ± 0.21	1.06 ± 0.20	1.40 ± 0.06 ^£^
		**22 ± 6**^#^*	**16 ± 3**^#^*	**2.44 ± 0.72**	**5.08 ± 1.38**^£^	**0.73 ± 0.09**^£^

### EPOR inhibition associated to radiotherapy or chemotherapy promotes senescence and mitotic death of glioma cells along with an increase of polyploidy and cyclin B1 expression

**Figure 4:**
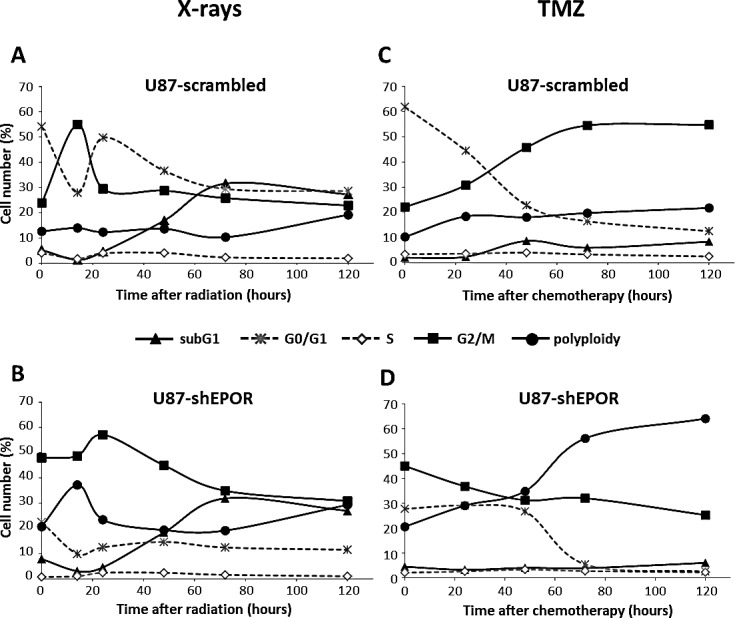
EPOR inhibition increases the polyploidy of U87 cells after radiation or temozolomide treatments U87 cells infected with scrambled shRNA or EPOR shRNA were treated with a single dose of X-rays (8 Gy) or TMZ (100 μM) 24 hours after seeding cells. At different times after treatment exposure (0h, 14h, 24h, 48h, 72h and 120h for X-rays and 0h, 24h, 48h, 72h and 120h for TMZ), cells were incubated with propidium iodide to realise a fluorescence-activated cell sorting (FACS)–based cell cycle analysis. Distribution kinetics of different cell cycle phases after X-rays treatment (8 Gy) and TMZ treatment (100 μM) of U87-scrambled (A and C, respectively) and U87-shEPOR (B and D, respectively). N=1 for each time point for both X-rays and TMZ treatments.

To study the mechanisms of EPOR down-regulation on radio- or chemosensitisation, we performed a flow cytometry study for U87-scrambled and U87-shEPOR cells following different times of treatment (from 0 to 120h) with either a single dose of X-rays (8 Gy) or TMZ (100 μM). As soon as 14h post-treatment, ionising radiation induce a transient accumulation of U87-scrambled cells in the G2/M phase, at the expense of cells of the G0/G1 phase (Figure 4A). This G2/M arrest is transient and followed by a shift of the cells in G1 phase at 24h post-radiation. When radiotherapy is combined with EPOR inhibition, glioma cells exhibit a similar cell proportion in the G2/M phase before and 14h after radiation (about 50% of cells). At this post-radiation time, a transient increase in polyploid cells is only observed for U87-shEPOR cells (U87-shEPOR=37% and U87-scrambled=13%) (Figure 4B). These results suggest that EPOR inhibition promotes polyploidy rather than potentiates the G2/M arrest as described for irradiated U87-scrambled cells. Then, a progressive accumulation of the cells in the subG1 phase is observed for the both cell types until 72h and maintained at 120h (Figures 4A and 4B). Of note, at 120h post-radiation, an increase in polyploid cells seems to start for both cell types (Figure 4A), but this effect is more pronounced for U87-shEPOR cells (Figure 4B). In response to EPOR inhibition combined to radiation, a biphasic increase in polyploid cells might be described in the acute phase after radiation (14h) followed by a late phase (120h).

Concerning the chemotherapy, G2/M arrest is not as early as observed for X-rays treatment but starts at 24h up to 120h post-TMZ (Figure 4C). When EPOR expression is knock-down, similar proportions of cells in G2/M phase, G0/G1 as well as in polyploidy were observed at 48h post-TMZ (Figure 4D). However, from 72h to 120h post-TMZ, although G2/M arrest persists or tends to slowly decline, the proportion of U87-shEPOR cells in polyploidy strongly increases concomitantly. Unlike X-rays treatment, for both U87-scrambled and U87-shEPOR, the proportion of cells in the subG1 phase is not changed 120h post-treatment with TMZ (Figures 4C and 4D).

**Figure 5:**
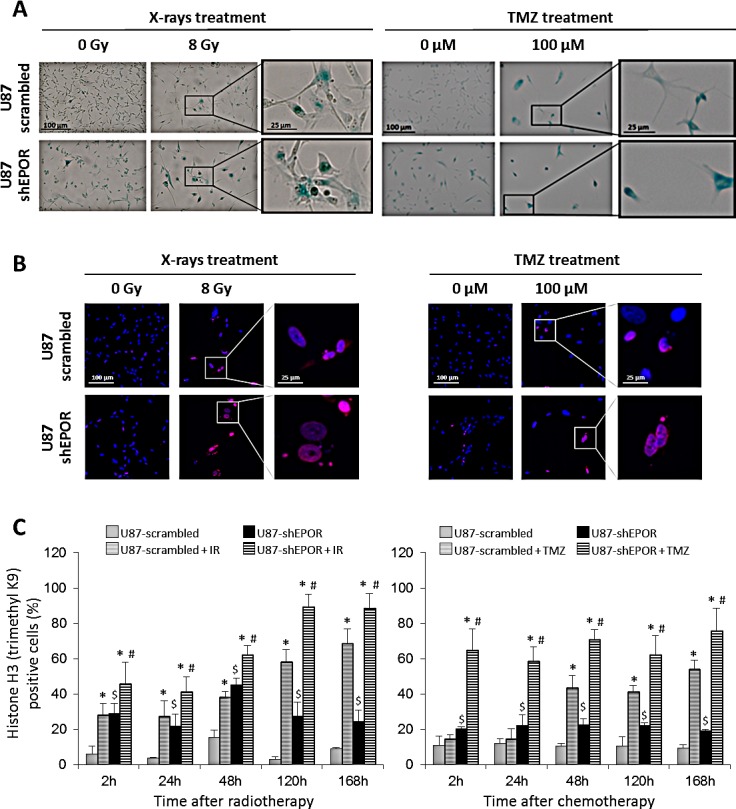
Senescence induced by X-rays treatment or temozolomide exposure is potentiated by EPOR inhibition on glioma cells (A) Representative photographs of senescence detected by β-galactosidase staining performed 5 days following a single dose of treatment (X-rays = 8 Gy or TMZ = 100 μM) on U87-scrambled and U87-shEPOR cells. The positive cells for senescence assay showed a blue coloration. Scale bar=100 μm for low magnification and scale bar=25 μm for high magnification. (B) Representative photographs of senescence detected by histone H3 (triMethylK9) immunostaining (red) on U87-scrambled and U87-shEPOR cells 5 days after X-rays (8 Gy) or TMZ (100 μM) treatments. Cell nuclei were identified with Hoechst 33342 staining (blue). Scale bar=100 μm for low magnification and scale bar=25 μm for high magnification. (C) Quantification of senescence on U87-scrambled and U87-shEPOR cells at different times (2h, 24h, 48h, 120h and 168h) after X-rays (8 Gy) (left graph) or TMZ (100 μM) (right graph) treatments. The proportion of histone H3 (TriMethylK9) positive cells was expressed relative to total cell number counted by Hoechst 33342 staining. Mean ± SD, n=9 (3 different experiments, 1 coverslip for each experiment, 3 representative fields per coverslip); * p<0.05 vs U87-scrambled or U87-shEPOR untreated for each cell type; $ p<0.05 vs U87-scrambled untreated and # p<0.05 vs U87-scrambled treated (Fisher's PLSD post-hoc test after a significant ANOVA).

All of these data indicate that cell cycle perturbations observed after treatment of shEPOR-glioma cells with radiation or TMZ are mediated by an abrogation of G2/M checkpoint leading mainly to polyploid cells. It suggests that the inhibition of EPOR expression might increase the cell death induced by radio- or chemotherapy in comparison to control cells. We next investigated the nature of the sensitisation to chemo- and radiotherapy induced by EPOR silencing by focusing on cell death especially cellular senescence. As depicted on Figure 5 and according to our previous cell cycle data (Figures 1 and 4), in control conditions, inhibition of EPOR expression increases the proportion of senescent cells compared to U87-scrambled cells (Figure 5A). We confirm that the X-rays exposure causes the senescence of control cells, but this phenomenon is amplified by the inhibition of EPOR since, proportionally, the number of blue cells is more important in U87-shEPOR group compared to U87-scrambled group. As described for the radiotherapy, chemotherapy-induced senescence is potentiated by EPOR down-regulation (Figure 5A).

**Figure 6:**
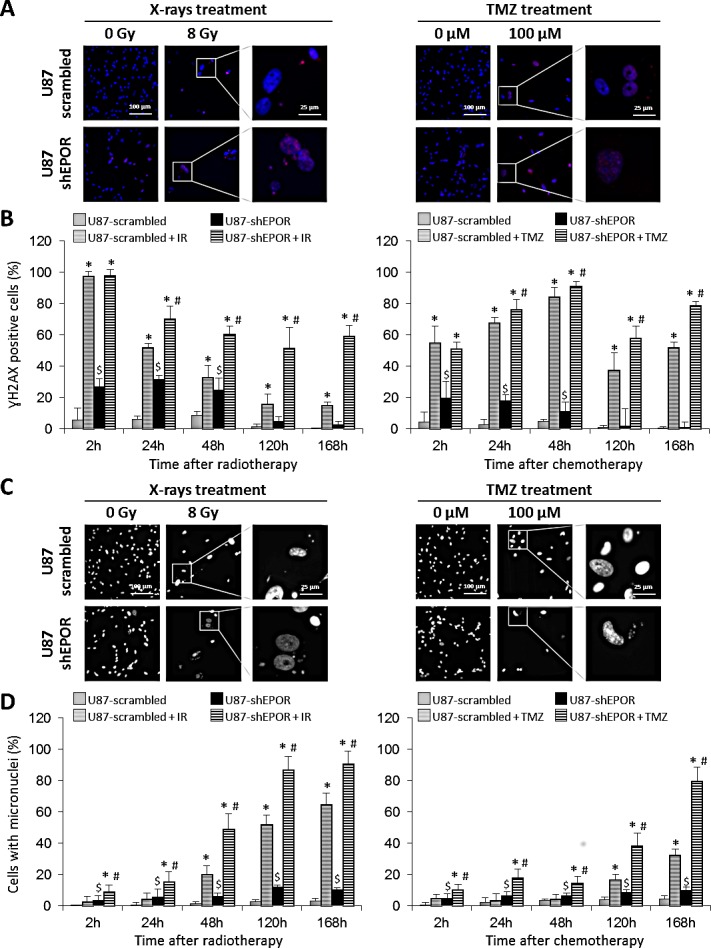
Mitotic death induced by X-rays treatment or temozolomide exposure is potentiated by EPOR inhibition on glioma cells (A) Representative photographs of DNA double-strand breaks identified with γH2AX immunostaining (red) to determine mitotic death on U87-scrambled and U87-shEPOR cells 5 days after a single exposure to X-rays (8 Gy) or TMZ (100 μM) treatments. Cell nuclei were identified with Hoechst 33342 staining (blue). Scale bar=100 μm for low magnification and scale bar=25 μm for high magnification. (B) Quantification of DNA breaks on U87-scrambled and U87-shEPOR cells at different times (2h, 24h, 48h, 120h and 168h) after X-rays (8 Gy) (left graph) or TMZ (100 μM) (right graph) treatments. The proportion of γH2AX positive cells was measured relative to total cell number counted by Hoechst 33342 staining. Mean ± SD, n=9 (3 different experiments, 1 coverslip for each experiment, 3 representative fields per coverslip); * p<0.05 vs U87-scrambled or U87-shEPOR untreated for each cell type; $ p<0.05 vs U87-scrambled untreated and # p<0.05 vs U87-scrambled treated (Fisher's PLSD post-hoc test after a significant ANOVA). (C) Representative photographs of genomic instability linked to mitotic death and identified by the presence of micronuclei on U87-scrambled and U87-shEPOR cells 5 days after a single exposure to X-rays (8 Gy) or TMZ (100 μM) treatments. Cell nuclei were identified with Hoechst 33342 staining (blue). Scale bar=100 μm for low magnification and scale bar=25 μm for high magnification. (D) Quantification of mitotic death evaluated by micronucleus assay on U87-scrambled and U87-shEPOR cells at different times (2h, 24h, 48h, 120h and 168h) after X-rays (8 Gy) (left graph) or TMZ (100 μM) (right graph) treatments. The proportion of positive cells (cell having at least one micronucleus) was obtained relative to total cell number counted by Hoechst staining. Mean ± SD, n=9 (3 different experiments, 1 coverslip for each experiment, 3 representative fields per coverslip); * p<0.05 vs U87-scrambled or U87-shEPOR untreated for each cell type; $ p<0.05 vs U87-scrambled untreated and # p<0.05 vs U87-scrambled treated (Fisher's PLSD post-hoc test after a significant ANOVA).

In order to strengthen these results, we studied others hallmarks of cellular senescence including the hypermethylation of a histone H3 (trimethylK9), that is related to senescence-associated heterochromatinization [57-59] and the persistence of gamma-H2AX foci [60,61]. As illustrated on Figures 5B and 6A, both treatments lead to the appearance of these senescence markers in control glioma cells exposed to X-rays and TMZ but these phenomena are amplified by the inhibition of EPOR. The quantitative kinetic analysis of histone H3 (trimethylK9) immunostaining shows that, regardless of the time after treatment, the proportion of positive U87-shEPOR cells is significantly higher than that of U87-scrambled cells (Figures 5B and 5C). Furthermore, this enhancement of histone H3 hypermethylation persists over time. Indeed, at 168h post-radiation: 88% of U87-shEPOR cells are still positive versus 68% for U87-scrambled cells (p<0.05) and at 168h post-TMZ, 76% of U87-shEPOR cells are still positive versus 54% for the U87-scrambled cells (p<0.05).

We also investigated the effects of the combined treatments on the DNA double-strand breaks by quantifying ɣH2AX positive cells after different times of radiotherapy or chemotherapy (Figures 6A and 6B). A soon as 2h post-radiation, all U87-scrambled and U87-shEPOR cells display DNA breaks (Figure 6B). However, for later times, for example at 168h post-radiation (Figure 6B), a large proportion of U87-shEPOR cells (59%) are still positive compared to U87-scrambled cells (15%, p<0.05). Similarly, we show a persistence of ɣH2AX foci after X-rays treatment (up to 168 hours) in the U87-shEPOR cells, a phenomenon that is not found in U87-scrambled cells (Figure 6B). Of note, this persistence of ɣH2AX foci, which reflects the genomic instability and the presence of irreversible senescence, is also observed in the U87-scrambled even after 168h of TMZ treatment (Figures 6A and 6B). However, the inhibition of EPOR amplifies this effect. At 168h post-TMZ, 79% of U87-shEPOR cells are still positive versus 52% for U87-scrambled cells (p<0.05) (Figure 6B). These results are in favour of a lack of DNA repair and might reflect a genomic instability.

One of the consequences of such genomic instability might be analysed by the presence of micronuclei [62-64]. The micronuclei formation analysed at various times after exposure glioma cells to radiation or TMZ (Figures 6C and 6D) corroborates the above results. Whichever post-irradiation time studied, the cell number with micronuclei is always higher in U87-shEPOR cells than those detected in U87-scrambled cells (Figure 6D) and at 120h and 168h after X-rays treatment, almost all U87-shEPOR cells display micronuclei. Similar results were obtained for TMZ treatment (Figure 6D), with a 2.5-fold increase of micronuclei in U87-shEPOR treated cells compared to U87-scrambled treated cells at 168h post-treatment.

Cells in senescence may return to the cell cycle but in most cases, they may undergo cell death via apoptosis or mitotic death [43]. Accordingly, we next addressed the question whether EPOR silencing might influence these cellular processes in response to X-rays or TMZ treatments. The increase in the subG1 populations observed for the irradiated U87 cells suggests the presence of apoptotic cells (Figures 4A and 4B).

**Figure 7:**
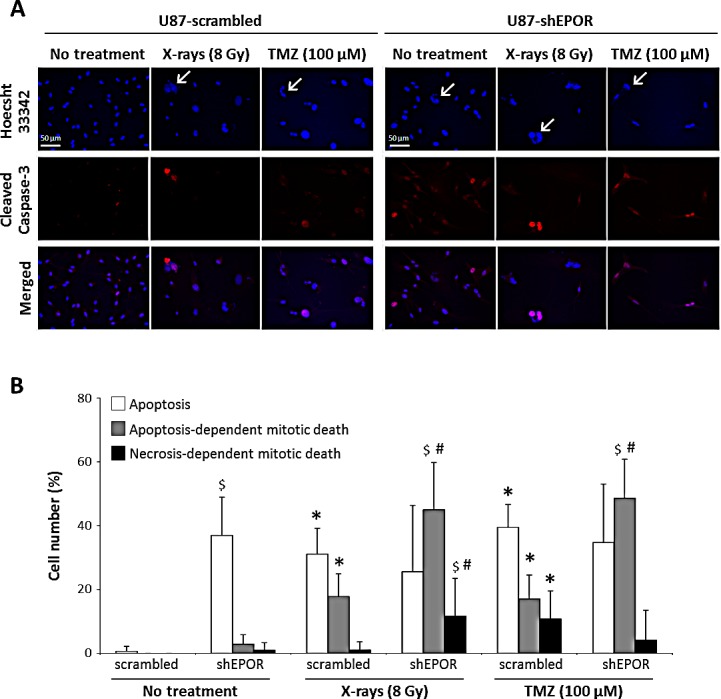
Apoptosis-dependent mitotic death induced by X-rays treatment or temozolomide exposure is potentiated by EPOR inhibition on glioma cells (A) Representative photographs of apoptosis by cleaved caspase-3 immunostaining (red) and mitotic catastrophe by multinucleated cells revealed with Hoechst 33342 staining (blue) on U87-scrambled and U87-shEPOR cells 7 days after a single X-rays (8 Gy) or TMZ (100 μM) treatments. The arrows denote the abnormal nuclei corresponding to cells in mitotic death and scale bar=50 μm. (B) Quantification of apoptosis and mitotic catastrophe 7 days after a single exposure to X-rays (8 Gy) or TMZ (100 μM) on U87-scrambled and U87-shEPOR cells. The cells with only one nucleus and positive for cleaved caspase-3 staining were identified as apoptotic (white bar). The cells with more than two nuclei and positive for cleaved caspase-3 were considered as cells in apoptosis-dependent mitotic death (gray bar). The cells with at least two nuclei and negative for cleaved caspase-3 were described as cells in necrosis-dependent mitotic death (dark bar). Mean ± SD, n=9 (3 different experiments, 1 coverslip for each experiment, 3 representative fields per coverslip); * p<0.05 vs U87-Scrambled untreated; # p<0.05 vs U87-shEPOR untreated and $ p<0.05 vs U87-scrambled for each treatment (Fisher's PLSD post-hoc test after a significant ANOVA).

Apoptosis induced by EPOR silencing is further confirmed by the activation of caspase-3, as demonstrated by the appearance of its cleaved active form (Figure 7A). Morphological examination of cells also reveals the presence of some large multinucleate cells either after X-rays and TMZ treatments or when glioma cells were depleted in EPOR (Figure 7A), indicating that some cells are entering a faulty mitosis without cytokinesis, corresponding to mitotic catastrophe. We next examined whether apoptosis or necrosis were involved in the final death of these cells. To discriminate apoptotic and necrotic cell death, cells showing multiple nuclei and a co-staining for cleaved caspase-3 were scored as cells in mitotic catastrophe committed to dying by a caspase-dependent way. A quantification of the cells dying by caspase-dependent (apoptosis-dependent mitotic death) or caspase-independent way (necrosis-dependent mitotic death) was performed (Figure 7B). From these results (Figures 7A and B), and according to cell cycle data (Figure 1B), EPOR silencing increases apoptosis of glioma cells. Indeed, under basal conditions, compared to U87-scrambled cells, 37% of U87-shEPOR cells are in apoptosis (p<0.0001) and 4% of these cells are in mitotic death (Figure 7B). Ionising radiation or TMZ significantly increases apoptosis as well as in mitotic death. However, although the population of apoptotic cells remains constant, sole the population of cells entering in mitotic death is amplified when the chemo- or radiotherapy were combined with EPOR inhibition (Figure 7B). In particular, 2.5 to 3 time more of U87-shEPOR cells in apoptosis-dependent mitotic death (p<0.05) are detected compared to control cells exposed to treatment alone (U87-scrambled cells treated with X-rays and with TMZ, respectively). EPOR inhibition enhances the overall glioma death in response to radiotherapy or chemotherapy (p<0.05) relative to scrambled cells, with a total of 83% versus 50% for irradiation, and 88% versus 67% for TMZ treatment (Figure 7B).

**Figure 8:**
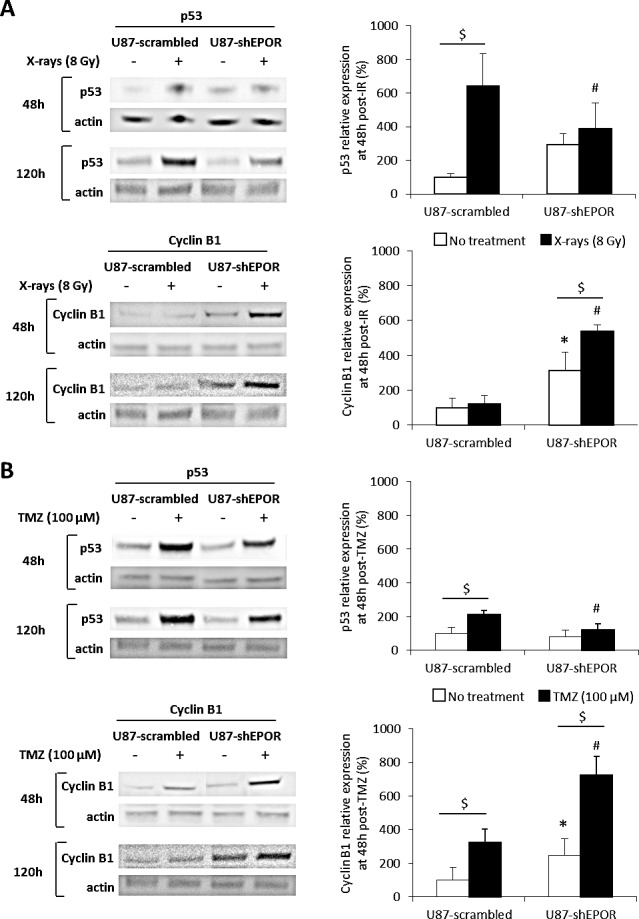
EPOR silencing on U87 cells increases cyclin B1 expression but does not modulate p53 expression induced by radiation or temozolomide exposures (A) U87 cells infected with scrambled shRNA or EPOR shRNA were treated or not with a single dose of X-rays (8 Gy) 24 hours after seeding cells. The total proteins were extracted 48 or 120 hours after radiation treatment to determine the protein expression of p53, an inducer of apoptosis, and cyclin B1, an effector of mitotic death. Cyclin B1 and p53 expressions were evaluated by western blot as shown the representative images obtained 48 and 120h after radiation exposure (left part) and the quantifications at 48 hours post-treatment, obtained by densitometry analyses (right part). Mean ± SD, n=3 for each cell type; $ p<0.05 for each cell type, * p<0.05 vs U87-scrambled untreated and # p<0.05 vs U87-scrambled + X-rays (Fisher's PLSD post-hoc test after a significant ANOVA). (B) U87-scrambled and U87-shEPOR cells were treated or not with a single dose of TMZ (100 μM) 24 hours after seeding cells. The total proteins were extracted 48 or 120 hours after drug treatment to determine by western blot the protein expression of p53 and cyclin B1 as shown the representative images (left part). The protein expressions of p53 and cyclin B1 were quantified by densitometry analyses 48 hours after TMZ treatment (right part). Mean ± SD, n=3 for each cell type; $ p<0.05 for each cell type, * p<0.05 vs U87-scrambled untreated and # p<0.05 vs U87-scrambled + TMZ (Fisher's PLSD post-hoc test after a significant ANOVA).

To strengthen the hypothesis that EPOR inhibition potentiates the mitotic death of cells subjected to radio- or chemotherapy, the levels of p53, as a major inducer of apoptosis, and of cyclin B1, as a molecular marker of mitotic death, were analysed by western blot 48h and 120h after treatments. Without treatment, EPOR down-regulation increases by 200% the expression of the cyclin B1 without any significant effect on p53 expression (Figures 8A and 8B). In response to X-rays, at 48h post-treatment, the expression of p53 is strongly induced in U87-scrambled cells compared to U87-shEPOR cells (U87-shEPOR+X-rays=389% versus U87-scrambled+X-rays=642%, p<0.05) (Figure 8A). In agreement with the results of cleaved caspase-3 immunostaining (Figure 7), 48h post-radiation, a robust increase in cyclin B1 expression is detected only in U87-shEPOR cells compared to U87-scrambled cells (U87-shEPOR+X-rays=538% versus U87-scrambled+X-rays=121%, p<0.05; Figure 8A) and importantly, this increase is sustained at 120h after radiation exposure. Similar results are observed for TMZ treatment (Figure 8B) with a higher level of p53 for U87-scrambled cells only and of cyclin B1 expression for U87-scrambled cells and U87-shEPOR cells. However, the increase of the cyclin B1 expression is more pronounced when chemotherapy is combined to EPOR inhibition (U87-shEPOR+TMZ=726% versus U87-scrambled+TMZ=325%, p<0.05) (Figure 8B).

## DISCUSSION

Glioblastoma (GBM) remains the most aggressive and lethal brain tumour due to its heterogeneity as well as high motility and invasion capabilities of its cells, resulting in high resistance to current standard treatments (surgery, followed by ionising radiation combined with temozolomide chemotherapy). Hypoxia, a main characteristic of GBM, is also generally associated with increased aggressiveness and resistance to the conventional treatments for these tumours [4,6,65]. The relative failure of the present treatment of GBM had encouraged developing alternative target therapeutic strategies such as anti-angiogenic therapies but with limited success [66]. One way to further improve the efficacy of conventional treatments might be to identify genes responsible for tumour resistance. Recently, we and others demonstrated that EPOR signalling on glioma cells, as well as on glioma stem cells, plays an important role in the tumour progression [19,21,22]. In this study, we determined, *in vitro,* that EPOR inhibition induces a G2/M arrest of tumour cells, consistent with our previous results showing that EPOR knock-down slows down the glioma growth. We also showed that EPOR silencing increases the sensitivity of normoxic and hypoxic GBM cells to TMZ and radiation treatments. The data presented here suggest that EPOR knock-down reinforces the efficacy of conventional treatments, by heightening the senescence that induces a robust genomic instability as evidenced by the presence of polyploid cells, the increase of cells with micronuclei and the persistent DNA breaks. This genetic instability combined to persistent increased of cyclin B1 expression finally leads to the caspase-dependent mitotic cell death of glioma cells.

Until now, controversial effects of EPO on glioma cells response to radiation or chemotherapy were reported. For instance, *in vivo* studies have shown that recombinant human EPO (rHuEPO) administration increases the efficacy of radiotherapy by reducing anaemia and tumour hypoxia [67,68]. In contrast, several studies indicated that an *in vitro* treatment with rHuEPO induces a significant resistance of glioma cells to ionising radiation and drugs which depends on signalling induced by EPOR [19,69,70]. Besides these studies with rHuEPO, our work represents the first investigation on the impact of the endogenous EPO/EPOR system present on glioma cells on the efficacy of radio- and chemotherapy. Interestingly, our results indicate that EPOR inhibition sensitises GBM cells to treatments in normoxia and hypoxia conditions. Interestingly, considering that hypoxia is a common feature of GBM, the observation that EPOR knock-down counteracts the hypoxia-induced chemo- and radioresistance is also noteworthy. These results were confirmed on a preclinical hypoxic GBM model. In this U251 model, EPOR silencing combined with TMZ treatment was more efficient to delay tumour relapse and to increase animal survival compared to TMZ alone.

We next aimed to identify the molecular and cellular features involved in the sensitisation of X-rays and TMZ induced by EPOR inhibition. In basal conditions, we show that the inhibition of EPOR affects the cell cycle of glioma cells with a G2/M arrest accompanied with an increase in the cyclin B1 expression, an indicator of G2/M arrest but not that of cyclin D1 expression, a critical mediator of G1 to S phase progression. In line with these results, EPOR knock-down inhibition leads to tumour cell apoptosis and premature senescence. These results are consistent with those described the past few decades, regarding the signalling pathways downstream of EPO/EPOR, which have been shown to influence anti-apoptotic pathways not only in erythroid cells but also in brain cells such as neurons [71,72]. A growing line of evidence identifies premature senescence as an important effector in response to chemotherapeutic agents as well as radiation and recently, it has been proposed that senescence-induced agents, when combined with other classic therapeutic approaches may be more effective in treatment of cancer [73]. Accordingly, therapy-induced senescence refers to a population of cancer cells that has been forced into senescence state by therapeutic agents. However, since cellular senescence might be reversible [43,74], the goal of these new therapeutic strategies is not only to force cancer cells to enter the senescence program but also to promote elimination of these senescent cells. Our results support this hypothesis. Indeed, we show that EPOR silencing in glioma cells forces the tumour cells treated with TMZ or ionising radiation toward senescence and mitotic catastrophe, reducing the proliferation capacities of glioma cells and, potentiating and prolonging the effects of the chemo- and radiotherapy. Collectively, our results suggest that inhibiting EPOR signalling on glioma cells might improve the first-line treatment of GBM. The current treatment was introduced in clinic assuming that TMZ sensitises GBM cells to radiation. Chemotherapy was expected not only to be a cytotoxic modality for malignant glioma but also, by producing a cell cycle arrest in G2/M phase, to sensitise the tumour cells to radiation [46,75]. It has been shown that TMZ sensitises GBM cells to radiation not through apoptosis, but through mitotic catastrophe induction [76,77]. Thus, treatments based on drug combinations that induce mitotic catastrophe leading to multiple mechanisms of cell death and growth arrest, deserve attention due to the efficacy to induce cell death. Accordingly, our results show that EPOR silencing on glioma cells increases the toxicity of TMZ and X-rays in GBM cells mainly through the enhancement of senescence leading to the induction of mitotic catastrophe.

Data of the present study argue in favor that in combination with X-rays or TMZ, silencing EPOR on glioma cells leads preferentially to a robust G2/M arrest which is accompanied by a robust genomic instability as evidenced by the presence of polyploid cells, the increase of cells with micronuclei as well as persistent DNA breaks. After several days of treatment, this senescence feature and the persistent high level of cyclin B1 expression is a scenario that produces cell death by apoptosis-dependent mitotic death. Our results are in concordance with recent studies showing a link between G2/M arrest and mitotic catastrophe through the regulation of cyclin B1 [40,77,78]. Collectively, these results suggest that mitotic cell death modulation strategy induced by EPOR inhibition could be considered as a therapy-induced senescence to reinforce the efficacy of standard treatments of GBM.

An increasing body of evidence identifies the therapeutically resistant and invasive subpopulation of cancer cells within GBM as tumour-initiating cells or glioma stem cells (GSC). Recently, according to our results, it has been proved that targeting EPOR expression decreased GSC tumorigenic potential [21] and GSC are known to be highly chemo- and radioresistant [79,80]. Accordingly, it could be important to evaluate whether silencing EPOR on these GSC could also improve the efficacy of conventional treatment on these pool of tumour cells.

## MATERIALS AND METHODS

### Cell lines

Two human glioblastoma cell lines were used: U87-MG (wild-type p53, [50,81]) purchased from American Type Culture Collections (ATCC, Manassas, VA, USA) and U251-MG (mutated p53, [50,81]) obtained from the National Cancer Institute (NCI, Bethesda, MD, USA). Tumour cells were cultured in DMEM (Sigma-Aldrich, France) supplemented with 10% foetal calf serum (FCS) (InVitrogen, France), 2 mM glutamine (Gln) (Sigma-Aldrich, France) and 100 U/ml penicillin/streptomycin (PS) (InVitrogen, France). Human UT7 cells, a megakaryoblastic leukemia cell line, were obtained from Dr. Patrick Mayeux (Institut Cochin, Paris, France). UT7 cells were cultured in alpha-modiﬁed Eagle's medium (Eurobio, France) with 10% FCS, 2 mM Gln, 100 U/ml PS and 2 IU/ml of rHuEPO (Proteogenix, France). All cell lines were maintained in culture at 37°C with 5% CO_2_ and 95% humidity and replated weekly.

### Hypoxia treatment

Hypoxia experiments were performed in a hypoxia chamber (*IN VIVO* 2 500, Ruskinn Technology and Awel International, France) set at 1% O_2_, 5% CO_2_ and 94% N_2_ at 37 °C. The culture medium was replaced by the same medium, equilibrated for 30 min with the gas mixture contained in the hypoxia chamber. The glioma cells were placed in the hypoxia workstation 18 hours after cell seeding (i.e. 6 hours before X-rays or drug treatments) and maintained under hypoxic or normoxic conditions (5% CO_2_ and 95% air at 37 °C) until the end of the experiment.

### Radiation and drug treatments

Glioma cells were exposed at room temperature to X-rays with doses ranging 0-8 Gy (Therac 15-Saturne with a dose rate of 2 Gy/min). All irradiation experiments were performed at the Radiotherapy Department of the CLCC (Centre de Lutte Contre le Cancer) François Baclesse (Caen, France) 24 hours after cell seeding.

Temozolomide (TMZ) prepared as a stock solution at 160 mM and stored at −20°C, was diluted in culture medium in presence of a concentration of DMSO (0.01%) that did not affect cell proliferation or survival. U87 and U251 cells were exposed to 25 μM or 100 μM TMZ which was brought in fresh medium.

### Lentiviral infection

Stable inhibition of EPOR expression in U87 and U251 cells was realised by RNA interference (Sigma-Aldrich, France). The cell infection by human EPOR shRNA was performed as described in previous study [22]. Control cells consisted of scrambled-shRNA infected cells.

### Clonogenic survival assay

For U251 cells, a standard clonogenic assay was used [82]. U251 cells were plated in 6-well plates (500 cells/ml), exposed to X-rays 24 hours after the cell seeding and kept in the incubator for duration of 10-14 days. Then, colonies were stained with 2% crystal violet (Sigma-Aldrich, France) diluted in 20% ethanol. Only colonies with more than 50 cells were counted manually. As the U87 cells do not form colonies on plastic [83], the soft agar assay for colony formation was used. In 6-well plates, 1 ml of top agarose solution (0.35% agarose) containing U87 cells (5000 cells/ml) was plated on 1 ml of base agar layer (0.5% agar). Standard medium (500 μl) is added in order to humidify the top agarose and is changed two times per week. U87 cells were exposed to X-rays 24 hours after the cell seeding and kept in the incubator for the next 21 days. The colonies were stained with 0.005% crystal violet (Sigma-Aldrich, France) diluted in 20% ethanol (Sigma-Aldrich, France) and counted [84]. For one experiment, the effect of each dose of radiation was studied on three individual wells of cell culture and each experiment was performed in quadruplicate.

The survival curves were analysed by a least square fit to the linear quadratic equation: SF = e-(αD + βD^2^) where α and β are the linear and quadratic parameters respectively; D corresponds to the absorbed dose and SF is the survival fraction. From the linear quadratic equations obtained with JMP software (SAS Institute Inc, Cary, NC, USA), several radiobiological parameters were calculated: SF2 (survival fraction at 2 Gy), D_0_ (mean lethal dose corresponding to dose for SF=37%), SER (sensitisation enhancement ratio determining by the ratio D_0_ control cells/D_0_ infected cells) and OER (oxygen enhancement ratio corresponding to the ratio D_0_ in hypoxia/D_0_ in normoxia).

### Proliferation assay

The cytotoxic effect of TMZ was evaluated by counting the cell number 72 hours after cell treatment in normoxic and hypoxic conditions. At the end of the treatment, U87 and U251 cells were fixed with 4% paraformaldehyde (PFA) (Sigma-Aldrich, France) in phosphate buffered saline (PBS) and cell nuclei were stained with Hoechst 33342 (10 μg/ml in PBS, Sigma-Aldrich, France). Photographs were acquired in a blinded style under fluorescence (4 wells per condition; 6 photographs per well) and the cell number was counted automatically with ImageJ (Rasband, W.S., ImageJ, U.S. N.I.H.). Each experiment was performed in triplicate.

With the same method, a cell proliferation study was performed to determine doubling time for each cell line according to the formula: T = t x ln2 × [ln(N2) - ln(N1)] with t = time between T1 and T2, N1 = the cell number at the time T1 and N2 = the cell number at the time T2. For all cells, the doubling time was calculated in the exponential phase of proliferation, in particular between days 2 and 3 after seeding.

### Cell cycle analysis

At various time points following cell exposure to X-rays (8 Gy) or TMZ (100 μM), cell cycle of U87 cells was studied by flow cytometry with Coulter DNA Prep Reagents kit according to manufacturer's instructions (Beckman Coulter SAS, France). Propidium iodide staining was analysed using FACSCalibur (Becton Dickinson Biosciences, France) with 20 000 events per determination. Analysis and determination of cell distribution in each phase of cell cycle was performed using the Cell Quest Pro software (Becton Dickinson Biosciences, France).

### Senescence assay

Senescence induction was determined based on alterations in cell morphology (enlargement and flattening) and expression of a pH-dependent β-galactosidase [29]. Senescence associated β-galactosidase staining (SA-β-gal) was realised as described by Dimri *et al*. [85] and according to manufacturer's instructions (Cell Signalling technology, USA).

### RT-qPCR analysis

RNA were withdrawn using Nucleospin® RNA II Kit (Macherey-Nagel, France) according to the manufacturer's protocol. One μg of total RNA from each sample was reverse-transcribed using the Promega RT system (Promega, France) (RT at 42°C for 1 h). Forward (F) and reverse (R) primers were designed for each gene using Beacon Designer software (Bio-Rad, France): human *EPOR*: F = 5′-CCTGACGCTCTCCCTCATCC-3′ and R = 5′-GCCTTCAAACTCGCTCTGTGG-3′; human *β-actin*: F = 5′-GACAGGATGCAGAAGGAGATTACT-3′ and R = 5′-TGATCCACATCTGCTGGAAGGT-3′. Assays were run in duplicate on the iCycler iQ™ real-time PCR detection system (Bio-Rad, France). The amplification profile was as follows: Hot Goldstar enzyme activation, 95°C for 3 min; PCR 50 cycles at 95°C, 15 sec and 60°C, 1 min. The PCR was done according to the manufacturer's protocol using the PCR™ Core Kit Sybr™ Green I (Eurogentec, France). The results were analysed using a comparative method between the fractional cycle number to reach a fixed threshold and the fractional cycle number of β-actin gene and expressed using the 2^−ΔCt^ formula.

### Western blot analysis

Cells, exposed or not for 48 or 120 hours to X-rays (8 Gy) or TMZ (100μM), were lysed with RIPA buffer (Sigma-Aldrich, France) supplemented with 1 μg/ml protease inhibitors (Sigma-Aldrich, France) and 1 μg/ml phosphatase inhibitors (Sigma-Aldrich, France). Proteins (40 μg) were separated by SDS-PAGE and transferred to polyvinylidene difluoride membranes (GE Healthcare Bio-Sciences, Sweden). The following primary antibodies were used: EPOR (1/200; Santa Cruz, M-20, sc-697), Cyclin D1 (1/2000; Cell Signalling Technology, DCS6, 2926S); Cyclin B1 (1/200; Santa Cruz, GNS1, sc-245) and p53 (1/200; Santa Cruz, DO-1, sc-126). β-actin antibody (1/5000; Sigma-Aldrich, A2066) was used to check the equal loading in proteins on the gel. Blots were exposed to peroxidase-linked secondary antibodies (anti-rabbit antibody: 1/10000; Sigma-Aldrich, A0545 and anti-mouse antibody: 1/5000; Sigma-Aldrich, A8924) and then the immunoreactive bands were visualised by enhanced chemiluminescence reagents (Thermo-Scientific, France). The band intensity of the different studied proteins was normalised to their corresponding actin signal and processed with ImageJ. Western blot photographs are representative of three independent experiments.

### Immunocytochemistry

Glioma cells were plated in 24-well plates on coverslips and one day later were exposed to X-rays (8 Gy) and TMZ (100 μM). At different times following treatments, cells were fixed with 4% PFA. Non-specific bindings were blocked with a solution of 3% bovine serum albumin (BSA) (Sigma-Aldrich, France)-PBS-0.1% Tween (Sigma-Aldrich, France) for 1 hour at room temperature. Then, cells were incubated overnight at 4°C with a primary antibody. The following primary antibodies were used: histone H3 (trimethylK9) (1/500; Abcam, ab8898); phospho-histone H2AX (ser139) (1/200; Cell Signalling Technology, D175, 2577S) and cleaved-caspase-3 (1/1600; Cell Signalling Technology, 9661S) in 1% BSA-PBS-0.1% Tween. The revelation was achieved by an Alexa-555-conjugated anti-rabbit secondary antibody (1/200; Molecular Probes, A21429). Cells were counterstained with Hoechst 33342 (10 μg/ml; Sigma-Aldrich, France) for nuclear staining. A micronucleus assay was performed from Hoechst 33342 staining and a cell with at least a micronucleus was considered positive. All immunocytochemical markers were observed on a Leica DM6000 microscope with a 40X objective. For each condition, at least 3 coverslips were analysed. Images from 3 representative high-power fields per slide were acquired.

### *In vivo* experiments

The animal investigations were performed under the European directive (86/609/EC). The licence to investigate was given to SV (14-55) in authorised housing and laboratories (B14118001) and with the permission of the regional committee on animal ethics (CENOMEXA, 0502-02). Mice were maintained in specific pathogen free housing and were fed with γ-irradiated laboratory chow and given water *ad libitum* (CURB, Central University of Caen Animal Care Facility).

Animals were manipulated under deep anaesthesia (5% isoflurane for induction, 2% for maintenance in 70% N_2_O/30% O_2_). Body temperature was monitored and maintained at 37.5±0.5°C with a feedback-controlled heating pad connected to a rectal probe.

Orthotopic glioma cells implantations were realised on male athymic *nude* mice (25-27 g; Charles River laboratory, L'Arbresle, France) according to our previous study [22].

TMZ (Temodal^TM^; Schering-Plough, France) was given *per os* (10 mg/kg/day on 5 consecutive days) when the tumours reached a volume of 30-40 mm^3^. Control animals received saline solution *per os*.

For determination of tumour volume, Magnetic Resonance Imaging (MRI) experiments were done once a week. MRI was performed on a 7 teslas horizontal magnet (Pharmascan, Bruker, Ettlingen). A cross coil configuration was used (volume/surface coil, Bruker, Ettlingen). The tumour was detected using an accelerated T2w sequence (RARE, acceleration factor of 8; TR/TE_eff_=5000/62.5 msec; number of experiments (NEX)=4; 20 contiguous slices; resolution=0.15×0.15×0.50 mm^3^; acquisition time=8 min). Tumour volumes were delineated manually with ImageJ.

### Data processing

All data are presented as mean ± SD. All statistical analyses were obtained using Student's *t*-test, Pearson-test for correlations and Fisher's PLSD post-hoc tests after significant ANOVAs with StatView SE (SAS Institute Inc, Cary, NC, USA).

## SUPPLEMENTARY MATERIAL AND FIGURES


