# Supplementary material for: Silencing erythropoietin receptor on glioma cells reinforces efficacy of temozolomide and X-rays through senescence and mitotic catastrophe
Source: Oncotarget. 2014 Dec 3;6(4):2101–19. doi: 10.18632/oncotarget.2937 (PMC4385839; doi:10.18632/oncotarget.2937)
Supplement: Supplementary file 1 [file oncotarget-06-2101-s001.pdf]

# Silencing erythropoietin receptor on glioma cells reinforces efficacy of temozolomide and X-rays through senescence and mitotic catastrophe

**Supplementary Material**

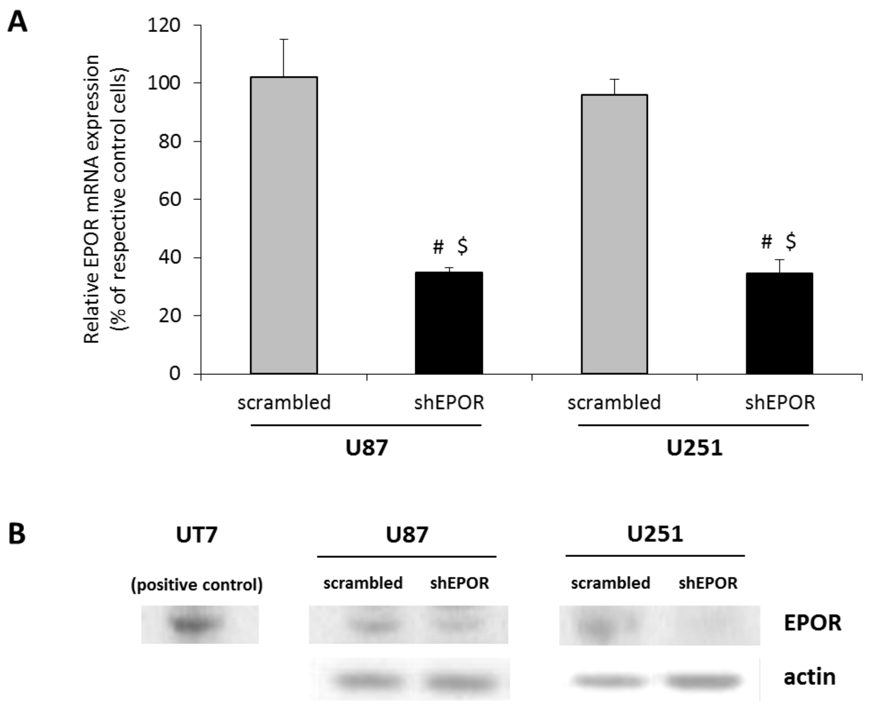


C

| Doubling time (hours) | U87 | U251 |
|---|---|---|
| control | 16.7 ± 2.2 | 15.3 ± 1.5 |
| scrambled | 16.9 ± 3.9 | 14.2 ± 1.7 |
| shEPOR | 24.0 ± 4.6 | 26.1 ± 3.2 |

**Figure S1: Determination of EPOR knock-down in human glioma cell lines and its impact of cell doubling time.**

Lentiviral infection was performed on U87 and U251 glioma cells to obtain a stable inhibition of EPOR expression by RNA interference. To ensure that the infection had no effect on glioma cells, scrambled shRNA were introduced in U87 and U251 cells.

**(A)** EPOR mRNA expression in U87 and U251 cells was determined by quantitative RT-PCR on scrambled and EPOR shRNA cells. The data are presented as relative expression to control cells, not infected. Mean ± SD,

n=3 different experiments; # $p<0.0001$ vs control cells and \$ $p<0.0001$ vs scrambled shRNA infected cells by a Fisher's PLSD post-hoc test after a significant ANOVA.

**(B)** Representative western-blot of EPOR in the scrambled and EPOR shRNA cells for both U87 and U251 glioma cell lines. Proteins withdrawn from the leukemia cell line, UT7 cells, were used as positive control for EPOR expression.

**(C)** Cell doubling time of control, scrambled and EPOR shRNA cells both U87 and U251 glioma cell lines. These data were obtained using the formula: $T = t \times \ln 2 \times [\ln(N2) - \ln(N1)]$ where t indicates the time between T1 and T2, N1 is the number cell at the time T1 and N2 is the number cell at the time T2. For all cells, the doubling time was calculated in the exponential phase of proliferation, in particular between days 2 and 3 after seeding. Mean ± SD, n=3 for each cell type.

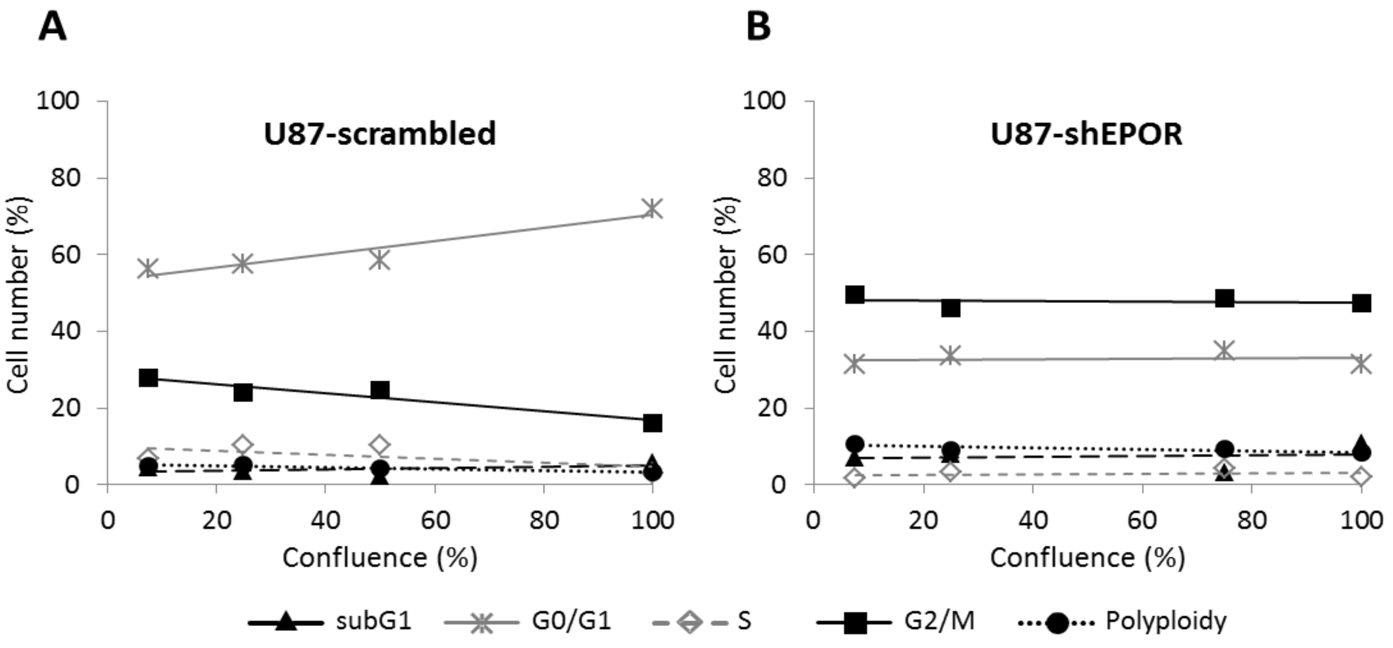


| U87-scrambled | | | | | |
|---|---|---|---|---|---|
| Confluence (%) | Cell number in each cell cycle phase (%) | | | | |
| | subG1 | G0/G1 | S | G2/M | Polyploidy |
| 7.5 | 4.62 | 56.10 | 6.61 | 27.77 | 4.89 |
| 25 | 3.43 | 57.36 | 10.13 | 23.91 | 5.17 |
| 50 | 2.17 | 58.62 | 10.36 | 24.57 | 4.29 |
| 100 | 5.84 | 71.86 | 2.89 | 16.07 | 3.34 |
| $R^2$ | 0.18 | 0.91 | 0.37 | 0.91 | 0.90 |
| **P value** | **0.64** | **0.06** | **0.46** | **0.07** | **0.06** |

| U87-shEPOR | | | | | |
|---|---|---|---|---|---|
| Confluence (%) | Cell number in each cell cycle phase (%) | | | | |
| | subG1 | G0/G1 | S | G2/M | Polyploidy |
| 7.5 | 7.15 | 31.20 | 1.49 | 49.49 | 10.67 |
| 25 | 8,00 | 33.65 | 3.25 | 46.08 | 9.02 |
| 50 | 3.28 | 34.74 | 4.25 | 48.42 | 9.31 |
| 100 | 11.04 | 31.39 | 1.93 | 47.44 | 8.20 |
| $R^2$ | 0.21 | 0.01 | 0.00 | 0.07 | 0.73 |
| **P value** | **0.62** | **0.91** | **1.00** | **0.79** | **0.20** |

**Figure S2: G2/M arrest induced by EPOR inhibition is not due to cell density of glioma cell culture.** U87-scrambled or U87-shEPOR cells were harvested to several times after seeding corresponding to different cell confluence (7.5, 25, 50 and 100%) and stained with propidium iodide to determine cell cycle status by flow cytometry. Correlation curves between the proportion in different phases of cell cycle and confluence of cell culture for U87-scrambled **(A)** and U87-shEPOR **(B)** cells (top part). All data are presented in table (bottom part) together with correlation coefficient $R^2$ and p-value (Pearson-test) for each cell cycle phase of U87-scrambled **(A)** and U87-shEPOR **(B)** cells. N=1 for each sample of U87-scrambled and U87-shEPOR cells.
